# Supplementary material for: Establishing need and population priorities to improve the health of homeless and vulnerably housed women, youth, and men: A Delphi consensus study
Source: PLoS One. 2020 Apr 16;15(4):e0231758. doi: 10.1371/journal.pone.0231758 (PMC7162520; doi:10.1371/journal.pone.0231758)
Supplement: S1 Appendix — (DOCX) [file pone.0231758.s001.docx]

Appendix A

Results of ranking of needs and populations after Delphi rounds 1 and 2.

Table 1: Needs identified as top priorities after Delphi round 1.

| Rank | Need | Vulnerably housed or homeless participants (%) | Health Professionals (%) | Total (%) |
| --- | --- | --- | --- | --- |
| 1 | Mental health and addiction care / trauma | 93.75 | 72.22 | 76.52 |
| 2 | Facilitating access to housing | 75 | 69.44 | 70.55 |
| 3 | Facilitating access to income support | 43.75 | 56.95 | 54.31 |
| 4 | Care co-ordination / case management | 37.5 | 53.52 | 50.32 |
| 5 | Chronic disease management (e.g. diabetes, smoking related lung disease) | 25 | 30.56 | 29.45 |
| 6 | End-of-life care (e.g. hospice, palliative care) | 6.25 | 11.11 | 10.14 |
| 7 | HIV, hepatitis B/C virus, and tuberculosis care | 18.75 | 6.95 | 9.31 |

Table 2: Needs identified as top four priorities after Delphi rounds 2 and 3.

| Rank | Need | Vulnerably housed or homeless participants (%) | Health Professionals (%) | Total (%) |
| --- | --- | --- | --- | --- |
| 1 | Facilitating access to housing | 90.91 | 96.92 | 95.35 |
| 2 | Mental health and addiction care / trauma | 87.88 | 96.92 | 93.80 |
| 3 | Care co-ordination / case management | 60.61 | 70.77 | 66.67 |
| 4 | Facilitating access to adequate income | 60.61 | 60.00 | 61.24 |
| 5 | Chronic disease management (e.g. diabetes, smoking related lung disease) | 22.73 | 36.92 | 30.23 |
| 6 | Nutrition and dietary support | 42.42 | 16.92 | 30.23 |
| 7 | HIV, hepatitis B/C virus, tuberculosis, and other infectious diseases care | 16.67 | 12.31 | 14.73 |
| 8 | End-of-life care (e.g. hospice, palliative care) | 10.61 | 13.85 | 12.40 |
| 9 | Exposure related illnesses | 13.64 | 1.54 | 7.75 |

Table 1: Populations identified as priorities after Delphi round 1.

| Rank | Need | Vulnerably housed or homeless participants (%) | Health Professionals (%) | Total (%) |
| --- | --- | --- | --- | --- |
| 1 | Indigenous (First Nations, Métis, Inuit) | 77.05 | 88.61 | 83.57 |
| 2 | People with acquired brain injury, intellectual, or physical disabilities | 78.69 | 65.82 | 71.43 |
| 3 | Youth | 73.77 | 65.82 | 69.29 |
| 4 | Women | 68.85 | 67.09 | 67.86 |
| 5 | Refugees | 32.79 | 49.37 | 42.14 |
| 6 | People with gender diversity (LGBTQ) | 27.87 | 26.58 | 27.14 |
| 7 | People with language barriers | 34.42 | 24.05 | 28.57 |

Table 2: Populations identified as top four priorities after Delphi rounds 2 and 3.

| Rank | Population | Vulnerably housed or homeless participants (%) | Health Professionals (%) | Total (%) |
| --- | --- | --- | --- | --- |
| 1 | Women, families and children | 75.86 | 81.03 | 79.31 |
| 2 | Indigenous (First Nations, Métis, Inuit) | 68.97 | 82.76 | 78.16 |
| 3 | People with acquired brain injury, intellectual, or physical disabilities | 72.41 | 62.07 | 65.52 |
| 4 | Youth | 44.83 | 68.97 | 60.92 |
| 5 | Refugees and migrants | 58.62 | 55.17 | 56.32 |
| 6 | Elderly | 37.93 | 65.52 | 56.32 |
| 7 | People with language barriers | 44.83 | 44.83 | 44.83 |
| 8 | Victims of intimate partner violence / domestic abuse | 31.03 | 50.0 | 43.68 |
| 90 | People with diverse sexual orientations and/or gender diversity (LGBTQ) | 24.14 | 48.28 | 40.23 |
| 10 | Visible minorities | 17.24 | 46.55 | 36.78 |
| 11 | Veterans | 13.79 | 43.10 | 33.33 |
