# Supplementary material for: Establishing need and population priorities to improve the health of homeless and vulnerably housed women, youth, and men: A Delphi consensus study
Source: PLoS One. 2020 Apr 16;15(4):e0231758. doi: 10.1371/journal.pone.0231758 (PMC7162520; doi:10.1371/journal.pone.0231758)
Supplement: S2 Appendix — (PDF) [file pone.0231758.s002.pdf]

Multi Stakeholder Delphi Consensus to Identify Priorities for Canadian Evidence-Based Guidelines to Improve the Health of Homeless and Vulnerably Housed People

This priority setting survey uses the Delphi consensus method to reach an agreement with homelessness health experts and those with lived experience of homelessness and their advocates, on important topics and special populations that may benefit from specific guidelines.

This Delphi includes three rounds of consensus building questions. After each round, you will get a short summary of expert opinions from the previous rounds for further prioritization. Rounds two and three will include specific sections targeting health experts and those with lived experience. **By participating in this Delphi, we ask that you respond in all three rounds.**

1. General Demographic Information

Please mark the best option. (Demographic information will be used in the analysis of options and priorities.)

1.1 My age is:

a. Less than 30 ☐
b. 31-40 ☐
c. 41-50 ☐
d. 51-60 ☐
e. 60 or more ☐

1.2 My gender is: :

a. Male ☐
b. Female ☐
c. Other ☐

1.3 My role in providing input is: (select the role/group that you feel you represent the most)

a. I am or have been homeless ☐
b. Primary care practitioner ☐
c. Specialist physician ☐
d. Registered nurse ☐
e. Public health expert ☐
f. Social worker ☐
g. Homelessness health researcher ☐
h. Community health advocate ☐
i. Other (please specify): ___________ ☐

1.4 I currently live or work in:

Please specify: __________________________ (City, Province)

1.5 My first language is:

English □

French □

Other □ (please specify) ________

1.6 At some time in my life, I have been homeless or vulnerably housed for: (we define vulnerably housed as living in poor-quality, temporary, or precarious type of housing, including single room hotels, shelters or rooming houses)

a. Less than 2 years □
b. 2-5 years □
c. 6-10 years □
d. 11+ years □
f. not applicable □

1.7 I have been involved in research/programs related to the health of homeless or vulnerably housed people for:

a. Less than 2 years □
b. 2-5 years □
c. 6-10 years □
d. 11+ years □
f. not applicable □

2. Core Values

2.1 Before we decide on guideline topics and develop the guidelines, we need to agree on a set of core values that will guide the development of the guidelines. Please rank the values listed below in order of importance, where one is the most important value and seven is the least important topic.

Value	**Rank the values (1 = highest importance to 7 = lowest importance)**
**Commitment to the right of each citizen to safe affordable housing**	☐
**Commitment to reducing the negative impacts from conditions in which people are born, grow, work, live, and age, and the systems that shape the conditions of daily life** (social determinants of health)	☐
**Commitment to universal access to health care services** (the ability of all people to have access to health care services)	☐
**Commitment to continuity of care**	☐
**Commitment to using evidence based methods for guidelines development**	☐
**Commitment to include in our processes people who have experienced homelessness**	☐
**Commitment to advocacy for the rights of people experiencing social disadvantage**	☐

2.2 Are there any other values that we should include?

2.3 Please provide feedback on the proposed core values.

3. Feedback on the focus of the homeless health guidelines

3.1 Below is a list of guideline topics/themes that we could develop to improve the health of homeless and vulnerably housed people in Canada. Based on your own experience, please consider these criteria when judging the importance of a topic:

- value added (opportunity for a unique and relevant contribution),
- equity (reducing unfair and preventable health inequalities),
- burden of illness (number of people that may suffer from a disease or condition).

In formulating your response, please consider that we have limited resources. We believe it is feasible to develop four to six core guidelines.

Please rank the topics in order of importance, with one being the highest and seven being the lowest.

	**Rank the priority topics (1 = highest importance to 7 = lowest importance)**
**Care co-ordination / case management**	☐
**Mental health and addiction care**	☐
**Chronic disease management (e.g. diabetes, smoking related lung disease)**	☐
**End-of-life care (e.g. hospice, palliative care)**	☐
**HIV, hepatitis B/C virus, and tuberculosis care**	☐
**Facilitating access to housing**	☐
**Facilitating access to income support**	☐

3.2 Are there any other guideline topics that we should include?

____

____

3.3 If applicable, please provide feedback on the guidelines topics from question 3.1. and 3.2.

____

____

____

____

____

3.4 Below is a list of groups of people that we want our guidelines to help. Our process included identifying groups that may require tailored approaches to ensure effective guidelines.

Please rate the importance of specific focus within the guidelines to reach the following groups, where one is the most important, and seven is the least important topic.

	Rank the groups (1 = most important to 7 = least important)
**Indigenous (First Nations, Métis, Inuit)**	☐
**People with language barriers**	☐
**People with acquired brain injury, intellectual, or physical disabilities**	☐
**People with gender diversity (LGBTQ)**	☐
**Refugees**	☐
**Women**	☐
**Youth**	☐

3.5 Are there any additional specific groups that we should consider?

____

____

3.6 If applicable, please provide feedback on approaches to reach the specific groups listed in questions 3.4 and 3.5

____

____

____

____

____

4. Refer an expert to the Delphi consensus

We want to have other experts and leaders in the health of homeless and vulnerably housed people. These leaders would join in the consensus survey process.

If possible, please refer up to two people who are experts in homeless health and who ***have agreed*** to be contacted by us to participate in the Delphi.

Name: ____
Contact phone: ____
Contact e-mail: ____

Name: ____
Contact phone: ____
Contact e-mail: ____

5. Refer a person who has experienced homelessness or is vulnerably housed to the Delphi consensus

We want to include the opinion of people who are or have been homeless to determine the guidelines topics and important sub-populations. These experts would join in the consensus survey process.

If possible, please refer a person who is or was homeless and who ***has agreed*** to be contacted by us to participate in the Delphi. This Delphi survey has been developed and piloted with people with lived experience; however, in order to optimize diversity with respect to literacy and language barriers, we ask that you be available to assist people with lived experience who need help completing the survey.

Name: ____
Contact phone: ____
Contact e-mail: ____

Thank you for taking the time to complete this survey! Your responses are greatly appreciated.
