# Supplementary material for: Establishing need and population priorities to improve the health of homeless and vulnerably housed women, youth, and men: A Delphi consensus study
Source: PLoS One. 2020 Apr 16;15(4):e0231758. doi: 10.1371/journal.pone.0231758 (PMC7162520; doi:10.1371/journal.pone.0231758)
Supplement: S3 Appendix — (PDF) [file pone.0231758.s003.pdf]

Consensus de Delphi multipartite visant à déterminer les priorités des lignes directrices canadiennes fondées sur des données probantes pour améliorer la santé des sans-abri et des personnes vulnérables

Cette enquête de définition des priorités utilise la méthode de consensus Delphi pour parvenir à un accord avec les experts en santé des sans-abri et ceux qui ont vécu l'expérience de l'itinérance et leurs défenseurs sur des sujets importants et des populations particulières qui pourraient bénéficier de directives spécifiques.

Ce Delphi comprend trois séries de questions de consensus. Après chaque tour, vous obtiendrez un bref résumé des avis d'experts des rondes précédentes pour une autre priorité. Les séries deux et trois comprendront des sections spécifiques ciblant les spécialistes de la santé et les personnes ayant une expérience vécue. **En participant à ce Delphi, nous vous demandons de répondre aux trois tours.**

1. Données démographiques générales

Veuillez indiquer la meilleure option. (L'information démographique sera utilisée dans l'analyse des options et des priorités.)

1.1 Mon âge est:

A. Moins de 30 ◻
B. 31-40 ◻
C. 41-50 ◻
ré. 51-60 ◻
E. 60 ou plus ◻

1.2 Mon genre est:

A. Mâle ◻
B. Femelle ◻
C. Autre ◻

1.3 Mon rôle dans la fourniture de l' entrée est: (sélectionnez le rôle / groupe que vous sentez que vous représentez le plus)

a. Je suis ou ai été sans domicile fixe ◻
b. Praticien de soins primaires ◻
c. Médecin spécialiste ◻
d. Infirmière autorisée ◻
e. Expert en santé publique ◻
f. Travailleur social ◻

g. Chercheur en santé sans domicile ☐

h. Défenseur de la santé communautaire ☐

i. Autre (s'il vous plaît préciser):__________ ☐

1.4 Je vis ou travailler en ce moment:

Veuillez préciser: ________________________ (Ville, Province)

1.5 ma première langue est:

Anglais ☐

Français ☐

Autres ☐ (s'il vous plaît préciser) ________

1.6 À un certain moment de ma vie, je suis sans abri ou vulnérablement logé pour: (nous définissons vulnérablement logés comme vivant dans la mauvaise qualité, temporaire, ou de type précaire de logement, y compris les hôtels simples dans la chambre, des refuges ou des maisons de chambres)

A. Moins de 2 ans ☐

B. 2-5 ans ☐

C. 6 à 10 ans ☐

D. 11 ans et plus ☐

F. n'est pas applicable ☐

1.7 j'ai Été impliqués dans la recherche / programmes liés à la santé des personnes sans - abri ou vulnérablement logés pour:

A. Moins de 2 ans ☐

B. 2-5 ans ☐

C. 6 à 10 ans ☐

D. 11 ans et plus ☐

E. n'est pas applicable ☐

2. Valeurs fondamentales

2.1 Avant de décider sur des sujets de lignes directrices et de développer les lignes directrices, nous avons besoin de se mettre d' accord sur un ensemble de valeurs fondamentales qui guideront l'élaboration des lignes directrices. S'il vous plaît classer les valeurs ci - dessous par ordre d'importance, où l' un est la valeur la plus importante et sept est le sujet le moins important.

	**Classez les valeurs (1 = importance la plus élevée à 7 = importance la plus faible)**
**Engagement envers le droit de chaque citoyen à** un **logement sûr** et **abordable**	☐
**Engagement à réduire les impacts négatifs des conditions dans lesquelles** les **individus naissent, grandissent, travaillent, vivent, et** l' **âge, et les systèmes qui façonnent les conditions de** la **vie quotidienne** (les déterminants sociaux de la santé)	☐
**Engagement envers l'accès universel aux services de santé** (La capacité de toutes les personnes d'avoir accès aux services de soins de santé)	☐
**Engagement envers la continuité des soins**	☐
**Méthodes basées** sur **des preuves d'engagement à u chanter pour** le **développement des lignes directrices**	☐
**Engagement à inclure dans le peuple de processus qui ont vécu** l' **itinérance**	☐
**Engagement à défendre les droits des personnes en situation de désavantage social**	☐

2.2 At - il d'autres valeurs que nous devrions inclure?

____

____

2.3 Bail fournir une rétroaction sur les valeurs fondamentales proposées.

____

____

____

____

____

____

3. Commentaires sur la mise au point des lignes directrices pour la santé sans - abri

3.1 Voici une liste de sujets / de thèmes que nous pourrions développer pour améliorer la santé des personnes sans abri et vulnérablement logés au Canada lignes directrices. Base de d sur votre propre expérience, s'il vous plaît tenir compte de ces critères pour juger de l'importance d'un sujet:

- valeur ajoutée (occasion pour a que uni et contribution pertinente),
- l' équité (en réduisant la santé injuste et évitable dans égalités),
- fardeau de la maladie (nombre de personnes qui peuvent souffrir d'une maladie ou affection).

En formulant votre réponse, veuillez considérer que nous avons des ressources limitées. Nous croyons qu'il est possible d'élaborer quatre à six lignes directrices.

Veuillez classer les sujets par ordre d'importance, où l'un est le sujet le plus important et quatre le sujet le moins important.

	Classer les sujets prioritaires (1 = importance la plus élevée à 7 = importance la plus faible)
**Coordination des soins / gestion de cas**	☐
**Soins de santé mentale et de toxicomanie**	☐
**Gestion des maladies chroniques (p. Ex. Diabète, maladies pulmonaires liées au tabagisme)**	☐
**Soins de fin de vie (P. Ex., Soins palliatifs)**	☐
**VIH, l'hépatite B / C virus, et les soins de la tuberculose**	☐
**Faciliter l'accès au logement**	☐
**Faciliter l'accès au soutien du revenu**	☐

3.2 Existe -t- il d'autres la ligne directrice des sujets que nous devrions inclure?

____

____

3.3 Le cas échéant, s'il vous plaît fournir une rétroaction sur les IRECTIVES sujets de la question 3.1. et 3.2.

____

____

____

____

____

3.4 Voici une liste des groupes de personnes que nous voulons que nos lignes directrices pour aider. Notre processus comprenait l'identification de groupes qui pourraient exiger des approches adaptées pour assurer des lignes directrices efficaces.

Veuillez évaluer l'importance d'une attention particulière dans les lignes directrices Pour atteindre les groupes suivants, où l'un est le plus important, et sept est le sujet le moins important.

	**Classement des groupes (1 = le plus important à 7 = le moins important)**
**Autochtones (Premières nations, Métis, Inuit)**	☐
**Les gens avec des barrières linguistiques**	☐
**Les personnes atteintes d'une lésion aiguë du cerveau, intellectuelle ou un handicap physique**	☐
**Les personnes atteintes de la diversité des genres (LGBTQ)**	☐
**Réfugiés**	☐
**Femmes**	☐
**Jeunesse**	☐

3.5 Y at - il des groupes spéciaux supplémentaires que nous devrions prendre en considération?

____

____

3.6 Le cas échéant, s'il vous plaît fournir une rétroaction sur les approches pour atteindre les groupes spéciaux énumérés dans les questions 3.4 et 3.5

____

____

____

____

____

4. Reportez - vous un expert pour le consensus Delphi

Nous voulons avoir d'autres experts et leaders dans la santé des sans-abri et des personnes vulnérables. Ces dirigeants se joindront au processus d'enquête par consensus.

Si possible, s'il vous plaît se référer à deux personnes qui sont des experts en matière de santé sans abri et qui ***ont accepté*** d'être contacté par nous pour participer à la Delphi.

Nom:	______________________________
Numéro du contact:	______________________________
Email du contact:	______________________________

Nom:	______________________________
Numéro du contact:	______________________________
Email du contact:	______________________________

5. Référer un personne qui a une expérience d'itinérance ou est vulnérablement logé au consensus Delphi

Nous voulons inclure l'opinion des personnes qui sont ou ont été sans abri pour déterminer les lignes directrices et les sous-populations importantes. Ces experts se joindront au processus d'enquête par consensus.

Si possible, s'il vous plaît se référer une personne qui est ou était sans abri et qui ***a accepté*** d'être contacté par nous pour participer à la Delphi. Cette enquête Delphi a été développée et pilotée avec des personnes ayant une expérience vécue; Cependant, afin d'optimiser la diversité en ce qui a trait à l'alphabétisation et aux barrières linguistiques, nous vous demandons d'être à votre disposition pour aider les personnes ayant une expérience vécue et qui ont besoin d'aide pour remplir le sondage.

Nom:	______________________________
Numéro du contact:	______________________________
Email du contact:	______________________________

Merci de prendre le temps de répondre à ce sondage! Vos réponses sont grandement appréciées.
