# Supplementary material for: Establishing need and population priorities to improve the health of homeless and vulnerably housed women, youth, and men: A Delphi consensus study
Source: PLoS One. 2020 Apr 16;15(4):e0231758. doi: 10.1371/journal.pone.0231758 (PMC7162520; doi:10.1371/journal.pone.0231758)
Supplement: Attachment — Submitted filename: Response letter PONE-D-19-19733.docx [file pone.0231758.s004.docx]

**PONE-D-18-14628 Detailed Response to reviewers**

**Review comments to authors**

Reviewer #1: The inclusion of PLE in this study is innovative and exemplary. However, I question whether the paper's findings are consequential enough for publication in PLOS. In addition, methods are poorly described and the discussion is strangely presented. Finally, implications for a wider readership are not discussed, nor are the potentially unique contributions of the DELPHI method with the inclusion of PLE.

**Response:** We thank the reviewer for their review and recognition of the innovative multi-stakeholder engagement in this study. However, we respectfully disagree on the significance of the results for PLoS. Modified Delphi informed evidence based research is currently guiding and improving health of refugees (Swinkels and Pottie CMAJ 2011), migrants (ECDC Migrant Health Guidance 2018), elderly (Farrell 2015 PLoS-Delphi) and now people experiencing homelessness. We have added this information into the methods section on page 5. We do agree there is a need to include more implication for a wider readership as homeless studies are becoming more widespread, for example the Campbell Collaboration has registered two reviews in the past six months, and an additional four have been proposed. We have further restructured and rewritten the discussion section to address more precisely the findings of our study.

(1) Briefly describe the Delphi method and include citations

**Response**: Thank you to the reviewer for pointing out the need to clarify our methodology. We have edited and revised our methods section on page 5, adding primary and secondary references to outline this method and in the context of vulnerable populations (Farrell PLoS 2015, Swinkels CMAJ 2011, Pottie CFP 2014).

(2) Explain the "purposeful snowball approach" to sampling

**Response**: We thank the reviewer for their interest. Recruitment of homeless and recently homeless persons, also called, persons with lived experience, required use of a strategic network of NGOs (foodbanks, shelters, drop in centres) who served this population, and given the challenges of developing trust in excluded populations, we used our Community scholars (paid team members with lived experience), the NGO leaders, and the persons with lived experience who had already participated to help us recruit others with lived experience. We collaborated with 10 centres from across Canada and were able to recruit 10—15 persons per centre to ensure some consideration of the diverse and transient living contexts. We have also further explained the sampling method and its relevance in reaching hard-to-reach populations on page 6 (Penrod *et al.*, 2003). While we acknowledge a sampling weakness of our approach, we considered the trade off of sampling only accessible and organized PLA versus sampling also the more transitional population.

(3) While inclusion of PLE is important, how do the authors think this affected the study outcomes?

Response: Thank you to the reviewer for your thoughtful observation. The Delphi Consensus process set out to prioritize conditions and populations in need of evidence based guidelines. Homeless persons are among the most disenfranchised and excluded populations. Offering an intervention may frequently be met with mistrust, and refusal (Magwood *et al.*, Cochrane Methods, 2018), and we also have conducted a qualitative systematic review that clearly shows this.

The inclusion of our PLE provided a complementary perspective to the expert practitioners. There were initial differences documented in conditions (mental health versus addiction) and priority populations (i.e. women versus youth) in the early waves of the Delphi, but with sharing of the results of both groups a consensus emerged. We thus obtained early agreement and validity for our priorities, something that cannot be underestimated in such work.

A significant concern revolves around inclusion of persons with lived experience as local experts in our Delphi group. While this multi stakeholder engagement approach is supported, there is a concern that these unconventional participants may reduce the validity of the priority setting results. There has never been evidence based guidelines developed for homeless and vulnerably housed people and we have not been able to find a list of published priority conditions for homeless people. Our previous literature reviews and expert experiences identified the challenge of uptake of interventions with homeless people. Barriers include lack of trust, lack of understanding of priority and need for inclusion in order to ensure a successful health equity approach (Magwood *et al.*, Cochrane Methods, 2018). We therefore took the important step of early inclusion and piloted the initial survey questions with a group of people with lived homelessness experience and we revised the survey accordingly. This group with lived experience were adamant that they do not want to be treated differently than the other experts who would be consulted for the study and that asking for their preferences in a different way, such as focus groups, would result in them being treated tokenistic rather than as full participants. Therefore, we ensured with their help that that the surveys would be appropriate for all participants. We detailed the challenges of recruitment, retention, loss to follow up and weaknesses in process. We analyzed the data using subgroup sensitively analysis. We also detail in the results the similar rankings and the evolution of that ranking. Most significantly we achieved inclusion, consensus and innovative multi stakeholder engagement to provide priority estimates to advise the systematic reviews on route to developing evidence based guidelines.

(4) Most of the text in results section repeats information in tables

**Response**: Thank you for this helpful comment. We have significantly reduced repetition and thus increase the readability of this paper.

(5) Presenting the discussion points as a table is odd, given the amount of text included. Looks like an outline

**Response:** We agree, this does not convey the discussion adequately. We have edited the discussion section, have removed the table and stay within the word count given the re-worked results.

(6) Results begin "we invited 190 people..." But later in the paragraph and in Figure 1, authors say they could not estimate how many PLE were invited to participate.

**Response:** We wish to emphasize that this was an external survey and the invitations to people with lived experience were conveyed by 10 NGOs across Canada. A total of 114 practitioners were invited of which 84 completed the first round of the Delphi. We also directly invited a portion- 15% of our persons with lived experience based on referrals and these persons participated, but as noted, we relied on our NGO partners to invite other PLE from their centres and we cannot truly estimate the gross number of homeless and vulnerably housed people invited. We have re-written the first sentence of the results section on page 7 accordingly.

(7) It's odd that Table 1 includes Round 1 and Round 3, but not Round 2

**Response:** We agree with the reviewer that a sense of continuum is needed in the results and we have added the results of Round 2 to the table.

(8) The discussion should include a synopsis of how these recommendations compare to previous recommendations, if applicable.

**Response:** Thank you to the reviewer for pointing this out. We did search for priority setting publications, however we know there are no evidence based guidelines for homeless populations at this point. We did find some expert opinion adapted clinical guidance work from the US on this topic and we have included their priorities in our discussion. The US topics, selected by medical team, were very disease focused, while our topics, selected by PLE and clinicians carried a mix of social determinant of health (ie housing) and disease (ie addiction). We have added this distinction.

(9) The issue of generalizability beyond Canada should be addressed in the discussion

**Response:** Yes, we agree this is an important omission. Working in collaboration the London based Campbell Collaboration we have actively collaborated with review teams in England, Northern Ireland and Norway and US. Registered Cochrane and Campbell systematic reviews for homeless populations have increased from 2 to 7 in past year. Evidence based clinical guidelines, the endpoint of our research, have not yet emerged outside of Canada. In summary we expect the systematic reviews to provide evidence internationally, and our Canadian guidelines to provide a catalyst for other evidence based guidelines.

Reviewer #3: The study utilized a revised Delphi consensus method to identify critical topics from providers and formerly homeless individuals that may inform evidence based guidelines development targeting homeless individuals/families. Such guidelines may be critical in promoting health among this especially vulnerable population. The followings are my comments that hope can be beneficial in improving the study:

1. What may the settings not suitable for human habitation as stated in the intro section? Abandoned buildings can be seen as settings not suitable for human habitation, but living there may be considered literal homelessness, rather than “hidden homelessness”.

**Response:** Thank you for your comment. The World Health Organization recently published a guideline that outlines housing conditions that are suitable or dangerous for people to live in. (WHO 2018). <https://www.who.int/sustainable-development/publications/housing-health-guidelines/en/>

We have provided a definition in the introduction to clarify this term and references and referenced WHO 2018. Our goal was to recognize vulnerably housed persons as well as homeless ones as they too may be important candidates for interventions who are facing comparable challenges.

2. The authors stated EB-Guidelines multiple times, however, do not specifically explain what EB-Guidelines may be and how such guidelines may be critical for health outcomes. A brief discussion or even short examples of EB-Guidelines in health promotion might be needed in the intro section.

**Response:** Thank you. We have added an explanation using a WHO example, and Cochrane Collaboration and our Equity Methods publications on page 5. Evidence-based medicine is an approach to medical practice intended to optimize [decision-making](https://en.wikipedia.org/wiki/Decision-making) by emphasizing the use of [evidence](https://en.wikipedia.org/wiki/Evidence) from well-designed and well-conducted [research](https://en.wikipedia.org/wiki/Research). It identifies evidence gaps and informs evidence based guidelines and policies.

Evidence based guidelines for marginalized populations can serve to frame the key conditions and develop systematic reviews (PICO questions) to assess the effectiveness, harms, cost, feasibility and acceptability of interventions. We follow the GRADE evidence to decision framework that required evidence on 10 different constructs to determine a recommendation for or against.

We have also used the same approach for deprescribing for other conditions and populations using a modified Delphi approach, including frail elderly (Farrell PLoS, 2015; Pottie and Farrell CFP, 2018), HIV, dental screening, depression, anemia, vision (Pottie CMAJ, 2011; Tugwell Method CMAJ, 2011; Swinkels CMAJ, 2011). We published the Canadian Evidence Based Guidelines for Immigrants and Refugees, which used the Delphi method to inform the GRADE Guidelines and has built a network of practitioners in Canada working on refugee health, informed gaps for research and policy, and led to adaptations for EU/EEA. Similar results have occurred with the PLoS ONE published Delphi for the Deprescribing guidelines and now network (Moriarty, Deprescribing recommendations: an essential consideration for clinical guideline developers. *Research in Social and Administrative Pharmacy, in press*).

3. Based on the title and the abstract, the Delphi consensus method seems to be a critical component in the current study. More detailed discussion regarding what Delphi consensus method is, the application of Delhi method in evidence based practices and the rationale of modification may be needed.

**Response:** We thank the reviewer and agree that a more detailed description may be helpful for readers and is feasible. We have modified the methods section (please also see response to point 2).

4. What may be the initial list that the authors generated by the experts?

**Response:** We have included this initial list of conditions in the appendices.

5. What domains/areas the literature that helps develop the initial list covers?
**Response:** As the reviewer can see from the appendix on list of initial conditions, these covered a range of conditions that we felt may be relevant and useful to investigate for homeless recommendations, such a homeless, TB, HIV, Hep C, Diabetes, etc. This initial list of topics was created based on a review of the literature, discussions among research team members with various academic and clinic backgrounds and in consultation with a group of 5 people with lived experience who also piloted the survey.

6. How were the experts selected when developing the initial list of topics? What might be the criteria?

**Response**: We used our homeless health network of prior connections and internet searches, we identified both junior and senior clinicians, researchers, policy makers, social workers and advocates in the field of homeless health and invited them to participate.

7. How did the modified Delphi method differ from the original method? What may be the rationale granting such modifications?

**Response:** The original Delphi methods was described by the RAND Corporation. In the last twenty years a modified Delphi method has become an industry standard. The Delphi method enables group, in-person, problem-solving using an iterative process of problem definition and discussion, feedback, and revisions. But the Modified Delphi Technique described here uses mail or email to gather information, provide feedback, and report conclusions.

We used the industry standard approach, however, we included PLE within our consensus process, and because this is a small departure from the more expert oriented approach, we documented their responses and conducted a sensitivity analysis on the PLE group. We acknowledge this approach is not conventional yet, as we argue above, we felt it was necessary to establish inclusion of people with lived experience.

8. In terms of the added unique challenges when conducting the Delphi survey, was this done in round 2 or round 3?

**Response:** We highlight several challenges primarily in the recruitment, retainment and collection of data for homeless people. Research with vulnerable populations, especially homeless persons faces challenges of trust, understanding and retainment in an unstable housing situation. We have mentioned these recruitment and retainment challenges as expected weaknesses in our Delphi. Including people who will be directly affect by the resulting evidence based guidelines with a sample from across the country is a strength as we were able to begin with an inclusive and open process.

9. What kind of data was collected (e.g., the participants simple rank the topics or can provide some comments?)? How the data were analyzed? More detailed information may be needed.

**Response:** Thank you to the reviewer for this comment. Ranking was a primary data and our survey provided space for other comments. We have added a more detailed description of the ranking analysis, and we used a qualitative analysis for the comments. When comments suggested other conditions or populations, these were inserted as possible conditions to be ranked in the following Delphi rounds.

10. Snowball sampling usually refers to using an initial set of participants to refer more participants who meet the participation criteria. I am not quite sure if this is the case in the study. It seems that the study recruit participants from some organizations, which sounds more like convenience sampling. More details regarding sampling may be needed. Also, how long was the sampling period?

**Response:** Yes, we agree this would be the definition of snowball sampling. We used our relationships with PLE to recruit them, and they helped recruit other PLE. To ensure we had a national sample we identified key NGOS to select and snowball sample persons with lived experience. We used a similar approach as our national team investigators new PLE at NGOs and this same approach was used to collect these geographically diverse participants. Sampling lasted from May to June 2017, which we added to the methods section on page 6.

11. It seems that table 1 does not showing Round two participant characteristics, what may be the rationale? Although the sample size might not allow the authors to conduct comprehensive analysis, I was wondering if the authors were able to conduct some basic analyses to see if there were differences between individuals who were able to complete the study vs. people who dropped out.

**Response:** The same participants were recruited in each of the rounds of the Delphi. Our intention of not outlining the characteristics from round 2 participants in the table was to keep the table focused. We agree that the reader could find the description of participants from each round important and we have redrafted the Table 1 to include Round 2 data. Our numbers are too small to illustrate statistically significant difference and the main differences between the characteristics or the first and last round respondents are outlined on page 9 of the manuscript. Our drop out rate was low, and reasons included family reasons, sabbatical, and work pressures. Reasons for PLE included limited access in mobile lifestyle.

12. This may go back to the analysis section or even the discussion of the modified Delphi procedures, in that how was the prioritization (i.e., importance) of topics determined? By accumulation of votes? What if there were ties in topics?

**Response:** Prioritization included an initial list based on the literature and feedback from a small group of clinicians, researchers and people with lived experience. Participants were asked to rank the topics on this list in order of importance during Delphi 1 survey, based on the ranking and additional written feedback. The list was modified and it was shared and ranked again, and then once more the ranking changed the list, and it was shared and ranked a third time. All participants received the results and all participants independently ranked conditions and groups.

The Delphi method is a structured communication technique, originally developed as a systematic, interactive forecasting method which relies on a panel of experts. Thus, experts are encouraged to revise their earlier answers in light of the replies of other members of their panel.

13. Would there be any steps to reconcile the differences between the two groups on the prioritization of topics? If no, then when developing EBI guidelines, at what domain which group’s prioritization should be taken into consideration?

**Response:** We believe EB guidelines are a social and networking enterprise. Our Delphi approach allowed us to engage patients and other stakeholders early in the development process to select priority needs that may benefit from guidelines. The science of the Delphi approach rest with the ranking and sharing of rank results so that participants are able to continue with their original rank or modify it when they see the results of other expert participants. In this way there is often some regression toward group priority topics. Ideally there is a consensus over 3 rounds that provides an estimate of priorities. This mediation between the preferences of all participants is an integral element of the Delphi process and the rankings of all participants had equal weight. All participants received the same three surveys. Within this mixed qualitative and quantitative approach participants provide rank and comments. While public health experts alone could pick the priorities, in the world of multi-stakeholder engagement we now know how important consumer and practitioner involvement can be when developing guidelines. In addition, a large team meeting took place following the conclusion of the Delphi process, which included both experts working in the field of homeless health and people with lived experience and the final ranked topics were again approved at that meeting (November 2017, Montreal).

14. Table 3 provides great information, regarding relevance of such topics on health promotion among homeless population. I was wondering if in the survey, participants were encouraged to provide rationale regarding their ranking of importance on the topics included in the survey.

**Response:** Participants were provided with a briefing within the survey and they were asked to provide comments and suggest missing conditions and populations. We are not aware of any Delphi Consensus requiring participants to provide a rationale for rankings, often ranking like most judgments are more of a gestalt, mixing different rationale. In the comment box, participants did not explain why they ranked the topics in a specific order. Considering that the background of participants has a potential impact on the topics they deemed most important, we ensured representation from different groups in terms of age, gender, profession and location. We did also provide all participants with a list of criteria to consider when ranking: (1) value added (opportunity for a unique and relevant contribution), (2) equity (reducing unfair and preventable health inequalities) and (3) burden of illness (number of people that may suffer from a disease or condition).
